# Tourette Syndrome as a Disorder of the Social Decision Making Network

**DOI:** 10.3389/fpsyt.2019.00742

**Published:** 2019-10-08

**Authors:** Roger L. Albin

**Affiliations:** ^1^GRECC & Neurology Service, VAAAHS, Ann Arbor, MI, United States; ^2^Department of Neurology, University of Michigan, Ann Arbor, MI, United States; ^3^University of Michigan Morris K. Udall Center of Excellence for Parkinson’s Disease Research, Ann Arbor, MI, United States; ^4^Michigan Alzheimer Disease Center, Ann Arbor, MI, United States

**Keywords:** tic, amygdala, periaqueductal gray, striatum, hypothalamus, cognitive control, emotional brain

## Abstract

Tourette syndrome is a common neurodevelopmental disorder defined by the presence of tics, stereotyped involuntary movements and phonations. Considerable evidence points to developmental abnormalities of the basal ganglia as tic substrates. Basal ganglia dysfunction does not account for important features of Tourette syndrome, including its natural history, male predominance, and the characteristic quality and distribution of tics. The latter mainly involve eye, face, and head movements, in addition to a variety of simple to complex phonations. A major normal function of these movements, and of phonations as well, is social signaling. Many important species- and sex-specific stereotyped social behaviors are mediated by a phylogenetically conserved network of subcortical nuclei, the social behavior network (SBN). Some SBN nuclei are sexually dimorphic, and SBN function is modulated strongly by gonadal steroids. Recent studies indicate that the SBN meshes with the basal ganglia to form a larger network, the Social Decision Making Network (SDM; O’Connell and Hofmann [2011]). The SDM concept overlaps significantly with Holstege’s (1993) model of an emotional motor system mediating socially relevant facial movements and phonations. Dopaminergic signaling within the basal ganglia component of the SDM may regulate social act motivation with the SBN component responsible for act expression. Developmental SDM abnormalities can explain all major Tourette syndrome features, including natural history, male predominance, the characteristic distribution of tics, and their stereotyped quality. Some data directly support this hypothesis. Tourette syndrome may be a disorder of social communication manifesting primarily as abnormal involuntary movements.

## Introduction

Tourette syndrome (TS) is a common neurodevelopmental disorder defined by characteristic stereotyped involuntary movements and phonations, tics, often accompanied by comorbid behavioral syndromes, particularly obsessive–compulsive disorder (1, 2). TS is usually conceptualized as a basal ganglia disorder (3, 4). This concept is supported by several lines of evidence, including clinical pharmacology, the presence of tics in other disorders with unequivocal basal ganglia pathology, and imaging data suggesting developmental abnormalities of the basal ganglia. Further strong support for basal ganglia dysfunction comes from experimental manipulations of basal ganglia pathways in non-human primates that produce credible tic analogues (5–8).

Basal ganglia dysfunction, however, does not obviously explain important features of TS. These include the natural history of TS with tic onset at ages 5–7, peripubertal exacerbations, and male predominance. Nor does basal ganglia dysfunction obviously explain the characteristic distribution of tics, which are usually eye, face, and head movements, nor the presence of involuntary phonations. Some recent imaging studies indicate widespread morphologic and functional abnormalities in the brains of TS subjects with both cortical and subcortical changes reported. This raises the possibility that TS is a meta-syndrome resulting from developmental abnormalities in any one or more of a variety of nodes in some relatively large brain network. This inference is consistent with recent genetic studies in TS, which implicate many genes in the pathogenesis of TS (9, 10).

Identification of a brain network whose abnormalities underlie TS should account for several features of TS:

1. The distribution of motor tics with the predominance of eye, facial, head, and shoulder movements
2. The presence and nature of vocal tics
3. The stereotyped nature of tics
4. The natural history of tics with incidence around ages 5–7 years, peripubertal exacerbation, and improvement with the transition to adulthood
5. Male TS predominance
6. Modulation of tic expression by attentional loading
7. Inclusion of the basal ganglia

These criteria also provide clues pointing us towards identification of the relevant network. The most important clue comes from considering a primary functional role of eye, face, and head movements—social signaling. Perhaps the major function of these movements is communication of emotional states between conspecifics. This aspect of non-verbal communication is important and universal among humans, with remarkably uniform use and interpretation of these kinds of movements (11). Darwin first suggested the social functions of these types of movements and argued that they are phylogenetically conserved across a wide variety of species (12). The concept that tics distort social signals helps to explain why TS and related tic disorders are generally perceived as disorders. Similar reasoning applies to vocal tics. The natural history of TS with tic onset around ages 5–7 and peripubertal exacerbations of tics suggests a role for gonadal steroids in tic expression, particularly androgens. The typical ages of tic onset coincide approximately with adrenarche, the period during which the adrenal cortex begins to produce the androgens dehydroepiandrosterone (DHEA) and DHEA sulfate (13, 14). Male predominance suggests that the involved brain network contains sexually dimorphic elements. Based on these clues, we are looking for a phylogenetically conserved brain network mediating social behaviors, driving the expression of stereotyped actions, strongly influenced by gonadal steroids, and containing anatomically or functionally sexually dimorphic elements.

## The Social Behavior Network, the Social Decision Making Network, and Holstege’s Emotional Motor System

In 1999, Newman proposed the existence of a pan-mammalian social behavior network (SBN) (15). In Newman’s original formulation, the SBN consists of the medial amygdala (MeA), the bed nucleus of the stria terminalis (BNST), the hypothalamic medial preoptic area (MPOA), the anterior hypothalamus (AH), the ventromedial hypothalamus (VMH), the lateral septum (LS), and the midbrain periaqueductal gray (PAG). These structures are densely interconnected, express high levels of gonadal steroid receptors, and are implicated in the control of numerous social behaviors, including mating, social affiliation, social submission, parenting, aggression, and defensive behaviors. These nuclei also express relatively high densities of receptors for the nonapeptides oxytocin and arginine vasopressin, both described as modulators of social behaviors (16). The hypothalamic paraventricular nucleus (PVN) is a major source of these nonapeptides, innervates SBN nuclei, and is considered by some to be a component of the SBN. Recent comparative studies indicate that the SBN is a conserved feature of the central nervous system of all vertebrates.

The SBN functions to integrate external and internal signals, the latter including gonadal steroids, to initiate and modulate social behaviors. The PAG is the efferent node of this network. Control of lordosis behavior, decoded by Pfaff and colleagues, is an excellent example of SBN function (17). Lordosis is the receptive posture adopted by female rats to facilitate mating. Lordosis is elicited by appropriate external stimulation and requires estrogen action within the VMH. VMH projections to the PAG modulate PAG control of lower brainstem nuclei responsible for generating this stereotyped but relatively complex motor act. A recent explosion of work using modern genetic and optogenetic methods indicates that complex social behaviors can be elicited by stimulation or inhibition of specific SBN nuclei and/or neuron subpopulations within SBN nuclei. Pup retrieval, for example, is elicited by stimulation of neurons within the MPOA (18). These behaviors are typically modulated by gonadal steroids in a sex-specific manner. Stolzenberg and Numan, for example, point out that similar pathways involving the MPOA and VMH produce different behavioral outputs in male and female mice (19). Gonadal steroid regulation of SBN nuclei gene expression is sex specific (20). Specific optogenetic stimulation of mouse MeA GABAergic neurons *in vivo* produces sexually dimorphic parenting (parenting by females, and by males at low stimulation rates) and anti-parenting (infanticide by males at high stimulation rates) behaviors. MeA GABAergic neurons exhibit significant transcriptome differences between male and female mice (21). These data indicate that some SBN nuclei are functionally sexually dimorphic, and anatomic sexual dimorphism of some SBN nuclei, notably the MPOA, was recognized decades ago (22).

**Figure 1:**
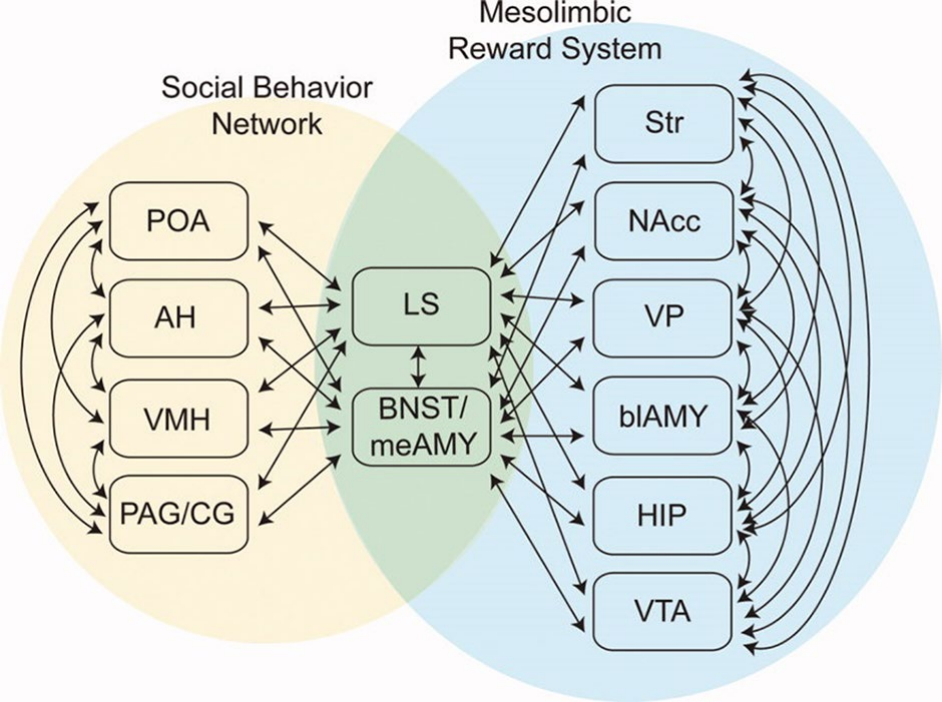
The Social Decision Making Network (SDM) model of O’Connell & Hofmann (23; reprinted with permission from John Wiley & Sons). The SDM combines basal ganglia circuits with the social behavior network (SBN) to form a greater network mediating evaluation of social signals, selection of appropriate social behaviors, and the initiation of relevant social behaviors. This cartoon omits direct projections from SBN hypothalamic nuclei to the VTA.

In 2011, O’Connell and Hofmann proposed an extension of the SBN concept to encompass forebrain nuclei involved in reward and motivation (23) (**Figure 1**). Added structures included the striatum (STR), nucleus accumbens (NA), ventral pallidum (VP), basolateral amygdala (BLA), hippocampal formation (HIP), and ventral tegmental area (VTA). O’Connell and Hofmann termed the expanded network the Social Decision Making Network (SDM) and presented a detailed comparative analysis suggesting that the SDM was phylogenetically conserved across all vertebrates (23). One recent re-evaluation of the SDM concept concluded that it is valid for mammals (24). General considerations about the nature of the human CNS also support the SDM concept. The large size and complexity of the human brain is suggested to be driven by requirements for complex analyses needed in social functioning (25). Close integration of phylogenetically ancient systems for evaluation of rewards and action outcomes (the basal ganglia) and social behaviors (Newman’s SBN) is likely critical for effective reproduction and functioning by highly social humans.

**Figure 2:**
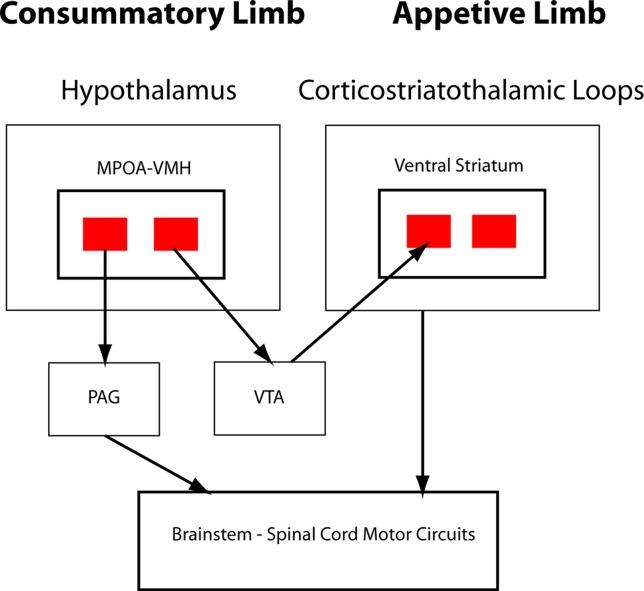
Schematic of hypothalamic–VTA interactions. Adapted from Stolzenberg and Numan (19; reprinted with permission from Oxford University Press). Two-limb model of the SDM for maternal and male and female sexual behaviors. VMH and medial preoptic area (MPOA) projections to VTA, with subsequent dopaminergic signaling in the ventral striatum, are responsible for evaluation of motivational significance of stimuli (appetitive behaviors). MPOA/VMH to periaqueductal gray (PAG) is responsible for initiating stereotyped social behaviors (consummatory behaviors).

In O’Connell and Hofmann’s formulation, critical interaction nodes between the SBN and basal ganglia components of the SDM are MeA, BNST (the MeA and BNST are generally conceptualized as linked components of the extended amygdala), and the LS (23). In their analysis of the circuitry underlying murine parental and sexual behaviors, Stolzenberg and Numan presented a complementary view by proposing direct projections from hypothalamic nuclei such as the MPOA and VMH to the VTA as critical for expression of social behaviors (19). In this formulation, the SDM has two limbs (**Figure 2**). There is a limb including the dopaminergic neurons of the VTA, the recipient ventral STR, and associated connections. Activity in this limb, influenced by hypothalamic nuclei (themselves modulated by gonadal steroids), is responsible for determining the relative value of rewards and actions, an important feature of VTA–ventral striatal regulation of motivational states. SBN outputs *via* the PAG are the second limb and are responsible for the actual performance of social behaviors via PAG modulation of downstream nuclei producing stereotyped motor acts. Recent evidence using sophisticated combinations of relevant behavioral assays, modern tract tracing methods, optogenetic manipulation of interconnections between hypothalamic nuclei and the basal ganglia, and gonadal steroid manipulations supports this model (18, 26–28). Dopaminergic neurotransmission, mainly involving the VTA–ventral striatal projection, is critical for expression of these stereotyped social behaviors. There may, however, be other relevant dopaminergic pathways. There is a significant projection from the VTA to the amygdala itself, a recently described nigrostriatal projection terminating in the LS (29), and dopaminergic neurotransmission occurs within several hypothalamic nuclei. Miller et al. recently presented data implicating dopaminergic VTA-to-MeA projections as mediators of approach–avoidance decision making in social contexts (30). MeA neurons receiving these dopaminergic inputs project to other SBN nuclei to regulate approach and avoidance behaviors.

The SDM concept overlaps other recent accounts of the “emotional brain” where emotions are conceptualized as functions critical to survival and reproduction (31). Most pertinent to TS, the SDM concept also overlaps with Holstege’s concept of an “emotional motor system,” which is a likely substrate for vocal tics (32–34). Holstege suggested the presence of two functionally parallel avenues for motor control. These include a voluntary motor system consisting of the traditionally identified corticospinal and corticobulbar pathways plus the medial descending systems (rubrospinal, reticulospinal, etc.) important for posture and gait. Complementing the voluntary system is the emotional motor system, which has access to lower motor and premotor neurons and can act independently of the voluntary system. A key node in Holstege’s model is the PAG, which modulates the activity of a number of more caudal nuclei responsible for the control of facial, oromandibular, lingual, and pharyngeal muscles, as well as pathways controlling respiration, diaphragm action, abdominal wall muscles, and pelvic floor muscles. Holstege particularly emphasizes the role of the emotional motor system in vocalization and speech production. In his model, the emotional motor system and the voluntary motor system normally collaborate, with the emotional motor system driving phonation and the voluntary system responsible for the precise modulation needed for human speech. Paralleling Holstege’s concepts is a model of the organization and evolution of human speech (35, 36). In this model of human speech control, there is a phylogenetically conserved primary vocal motor network (PVMN), including the PAG (and downstream brainstem circuits), the amygdala, and the hypothalamus, and a superimposed volitional articulatory motor network (VAMN), including the ventrolateral prefrontal cortex (Broca’s area), premotor cortex, and primary motor cortex. In almost all mammals but humans, vocalization content is emotional in content, and the emotional motor system—PVMN—is the primary pathway for vocalization. In both cats and non-human primates, PAG stimulation, even in decerebrate preparations, produces natural-sounding vocalizations (37). In non-human primates, amygdala stimulation produces a full range of natural vocalizations (38). The emergence of the VAMN is necessary for learned, symbolically rich speech-language. Its evolution from pre-adaptations is suggested to follow traditional concepts of expanding prefrontal cortices and increasing importance of cortiobulbar projections from primary motor cortex to motor neurons (36). In normal circumstances, the VAMN exerts both top–down hierarchic modulation of the PVMN and parallel direct modulation of brainstem motor circuits. Independent activity of the emotional motor system occurs in some human clinical situations and is a plausible explanation for vocal tics (32).

The SDM concept can also plausibly incorporate the common phenomenon of attentional loading modulating tic expression. In an interesting synthesis, Bickart et al. suggested that different amygdalar nuclei are components of overlapping brain networks involved in different aspects of social behavior (39). Bickart et al. point out that both the MeA and BLA are also components of the brain default mode network, whose activity is modulated strongly by attention and task engagement. If these amygdalar nuclei are important nodes in a disturbed network that generates tics, then changes in MeA–BLA activity accompanying changing default mode network function are a plausible explanation for task engagement affecting tic expression.

The SDM concept also offers a potential explanation for the frequent amelioration of tics as TS subjects enter adulthood. Recent studies of the developmental trajectory of emotional regulation in humans emphasize the key roles of functional changes in components of the SDM, notably the amygdala and VS [summarized nicely by Casey et al. (40)]. Functional connectivity and task-related Magnetic Resonance Imaging (MRI) studies indicate a dynamic relationship in the functional relationship between the amygdala and VS, correlated with performance on tasks reflecting emotional maturity, from adolescence into adulthood. By some measures, the maturation of the emotional brain isn’t complete until the mid-20s. These studies also point to increasing importance of descending control from frontal cortices in maturation of the emotional brain. Casey et al. present a sophisticated model of the childhood, adolescence, and adulthood emotional brain developmental trajectory as characterized by serial, hierarchal changes in subcortical circuits, followed by changes in corticosubcortical circuits and then corticocortical circuits (40). The amygdala is a particularly important node in this model. The natural history of TS fits well with this general concept of the developing emotional brain and specifically with developmental changes in the interactions of the amygdala and Ventral Striatum (VS), and increasing cognitive control from descending cortical inputs to these structures during the transition to adulthood.

## Retrodictions

As described above, developmental abnormalities of the SDM can theoretically account for several major features of TS. The SDM hypothesis can also account for a number of other observations and findings related to TS. If TS is a disorder of a network critical to social behavior, then we’d expect to find other evidence of social dysfunction in TS subjects. This is now a well-documented aspect of TS. The most prominent example is the rare phenomenon of coprolalia. Many individuals with TS manifest other socially inappropriate behaviors, originally described by Kurlan as non-obscene socially inappropriate symptoms (NOSISs; more properly non-obscene socially inappropriate behaviors—NOSIBs) (41, 42). There is also evidence of altered social perceptions in TS (43).

A number of studies investigated social cognition in TS, focusing on Theory of Mind (ToM)–related functions such as mirroring and mentalizing [reviewed comprehensively by Eddy (44)]. Some of these studies include results consistent with the SDM hypothesis. An interesting set of results suggests that TS subjects tend to over-interpret aspects, particularly potentially negative aspects, of social interactions—a hyper-mentalizing tendency. The presence of hyper-mentalizing in TS is supported by experiments with a paradigm where subjects viewed animations of moving triangles. TS subjects tended to attribute intentionality to randomly moving triangles (45). Subsequent task-related MRI studies using different paradigms to probe ToM in TS revealed differences between TS and control subjects in activation of several cortical regions, notably the right temporo-parietal junction, but also in the right amygdala (46, 47). Eddy et al. suggested that one potential substrate for tics is inappropriate reactivity of the amygdala to environmental cues (47).

Some imaging data support abnormalities of SDM-associated nuclei in TS. Using [^11^C]flumazenil positron emission tomography to image brain GABA-A receptors, Lerner et al. described abnormal ligand binding in the VS, amygdala, and PAG (48). MRI morphometry studies document amygdala abnormalities, and complementing morphologic studies of the amygdala is some work on amygdala function in TS (49, 50). A well-characterized aspect of amygdala function is participation in recognition of facial expression emotional content. Using task-related MRI, Neuner et al. reported that TS subjects exhibit deficits in these kinds of tasks and that the behavioral abnormalities are associated with abnormal amygdala activity (50). Garraux et al. reported abnormal midbrain morphology in TS subjects, consistent with PAG abnormalities (51). PAG involvement in TS was initially suggested by Devinsky in the early 1980s (52). He pointed to a potential pathologic correlate in encephalitis lethargica, whose victims apparently frequently exhibited tics. PAG pathologic changes were a hallmark of this now-vanished disorder. The most direct imaging evidence supporting the SDM hypothesis is recent imaging work performed by Greene et al. Using MRI voxel-based morphometry in a large sample of children with TS, this group demonstrated volumetric abnormalities of the PAG and hypothalamus (49).

It is important to recognize some potentially discrepant data. Imaging research also suggests the involvement of other regions. Greene et al., for example, documented volumetric changes in the pulvinar, a thalamic structure thought to be involved in modulation of visual attention (49). There are also a number of reports of anatomic and/or functional neocortical abnormalities in TS (46, 47, 53–55). Some of these data can be incorporated into the SDM hypothesis. As pointed out by Vicario and Martino, impaired structure or function of frontal cortical structures such as the insular cortex and the ventromedial prefrontal cortex is altered in TS, and these structures are implicated in social functioning (56). As described by Casey et al. in their model of emotional brain development, these are structures whose effects on relevant behaviors may be mediated by modulating components of the SDM such as the amygdala and VS (40). A speculative but plausible model would be some form of mistiming between development of SDM components and relatively late-maturing neocortical regions important for top–down modulation of SDM functions. Mistiming in development of connections between the SDM and relevant neocortical networks is a similar hypothesis.

## Implications

The SDM hypothesis offers a framework for further study of TS. Specific hypotheses, for example, about discrepant timing in the development of the SDM and relevant neocortical networks, could be evaluated with increasingly powerful imaging methods. Focusing on vocal tics might be particularly fruitful in this context because of the relatively advanced delineation of networks underlying human speech and their apparent division into the PVMN, which overlaps considerably with the SBN, and the neocortically based VAMN.

The SDM hypothesis may open an avenue to development of a circuit and pathophysiologic-based rodent model of tics. Very recent data indicate that the circuits underlying social vocalizations in mice are homologous to those described in other mammals, likely including homology with the human PVMN (57). Existing models, both non-human primate models and derivative rodent models (8, 58), are based on acute pharmacologic manipulation of basal ganglia circuits. The non-human primate models possess good face validity. Both the non-human and rodent models possess a degree of construct validity, as they are based on basal ganglia circuit manipulations. Non-human primate models are expensive and available only to a small number of investigators. A faithful rodent model would be very useful. Manipulation of the phylogenetically conserved circuitry underlying murine social vocalizations may allow development of a tic model with a greater degree of construct validity. An ideal rodent model would mimic not only the human PVMN but also the human VAMN. Okbi et al. recently described an apparently unique murine homologue of the human VAMN in Alston’s singing mice, a neotropical species with distinctive social vocalizations (59). Indeed, it is possible that Alston’s singing mice are the only other mammal with a corticobulbar projection system analogous to the human VAMN. Manipulation of the circuits underlying social vocalizations in Alston’s singing mice may provide a construct-valid, and ultimately predictively valid, model of tics.

The majority of children with TS have mild-moderate tics and behavioral comorbidities. Many of these children do not require treatment, particularly in view of the relatively benign natural history. Reassurance and a supportive social environment are often sufficient. Others may benefit from behavioral interventions, several of which have a solid evidentiary base. There are both children and adults, however, with troublesome tics and behavioral comorbidities. Conventional pharmacotherapies often have limited efficacy and/or limiting side effects, and while some may benefit from deep brain stimulation, there is a need for improved therapies. The SDM hypothesis suggests novel targets. Two nodes of the SDM, the amygdala and the PAG, may be worth particular focus. Conceptualizing TS as a disorder of social communication, the critical role of amygdala nuclei in social behaviors suggests that modulating amygdala function would address a basic pathophysiologic feature of TS. Focusing more specifically on tics, the PAG, as the critical SDM output node regulating motor pathways, is an attractive intervention target.

These considerations underscore the potential utility of the concepts and knowledge emerging from the burgeoning field of social neuroscience. These include both the detailed neurobiology emerging from the (mainly murine) studies of the SBN and the broader perspectives from human developmental studies of the emotional brain and social cognition. Reciprocally, studies of TS in these intellectual contexts may be helpful in understanding the neurobiology of human social behavior.

Finally, TS may be an interesting example of a more general neurologic phenomenon. Daniel Wolpert pointed out that movement is the primary way that animals influence their environment, leading to his suggestion that nervous systems exist primarily to generate and control movement (60). A corollary of this intriguing idea is that abnormalities of brain systems not directly involved in motor control manifest primarily as motor dysfunctions. The core feature of parkinsonism, bradykinesia, is likely an example of Wolpert’s corollary. A substantial body of theory and empirical evidence indicates that bradykinesia results from deficient motivational function with nigrostriatal dopaminergic deficits resulting in impaired scaling of actions to perceived action outcome values (61, 62). In the case of TS, dysfunction of the social communication system presents as involuntary movements.

## Author Contributions

RA is responsible for all aspects of this manuscript.

## Funding

This article was supported by NIH-NINDS R21NS088302, P50NS091856, and NIH-NIA P30AG053760.

## Conflict of Interest

The author declares that the research was conducted in the absence of any commercial or financial relationships that could be construed as a potential conflict of interest.

## Abbreviations

MPOA, medial preoptic area; AH, anterior hypothalamus; VMH, ventromedial hypothalamus; PAG, periaqueductal gray; LS, lateral septum; BNST, bed nucleus of the stria terminalis; meAMY, medial amygdala; Str, striatum; NAcc, nucleus accumbens; VP, ventral pallidum; blAMY, basolateral amygdala; HIP, hippocampal formation; VS, ventral striatum; VTA, ventral tegmental area.
